# Nodulation in *Dimorphandra wilsonii* Rizz. (Caesalpinioideae), a Threatened Species Native to the Brazilian Cerrado

**DOI:** 10.1371/journal.pone.0049520

**Published:** 2012-11-19

**Authors:** Márcia Bacelar Fonseca, Alvaro Peix, Sergio Miana de Faria, Pedro F. Mateos, Lina P. Rivera, Jean L. Simões-Araujo, Marcel Giovanni Costa França, Rosy Mary dos Santos Isaias, Cristina Cruz, Encarna Velázquez, Maria Rita Scotti, Janet I. Sprent, Euan K. James

**Affiliations:** 1 Depto de Botânica, Universidade Federal de Minas Gerais, Belo Horizonte, Minas Gerais, Brazil; 2 Instituto de Recursos Naturales y Agrobiología, IRNASA-CSIC, Unidad Asociada Universidad de Salamanca-CSIC ‘Interacción Planta-Microorganismo’, Salamanca, Spain; 3 Embrapa Agrobiologia, Seropédica, Rio de Janeiro, Brazil; 4 Departamento de Microbiología y Genética-CIALE, Universidad de Salamanca, Unidad Asociada Universidad de Salamanca-CSIC ‘Interacción Planta-Microorganismo’, Salamanca, Spain; 5 Universidade de Lisboa, Faculdade de Ciências, Centro de Biologia Ambiental (CBA), Lisboa, Portugal; 6 Division of Plant Sciences, University of Dundee at James Hutton Institute, Dundee, United Kingdom; 7 James Hutton Institute, Dundee, United Kingdom; University of California, Berkeley, United States of America

## Abstract

The threatened caesalpinioid legume *Dimorphandra wilsonii*, which is native to the Cerrado biome in Brazil, was examined for its nodulation and N_2_-fixing ability, and was compared with another, less-threatened species, *D. jorgei*. Nodulation and potential N_2_ fixation was shown on seedlings that had been inoculated singly with five bradyrhizobial isolates from mature *D. wilsonii* nodules. The infection of *D. wilsonii* by two of these strains (Dw10.1, Dw12.5) was followed in detail using light and transmission electron microscopy, and was compared with that of *D. jorgei* by *Bradyrhizobium* strain SEMIA6099. The roots of *D. wilsonii* were infected via small transient root hairs at 42 d after inoculation (dai), and nodules were sufficiently mature at 63 dai to express nitrogenase protein. Similar infection and nodule developmental processes were observed in *D. jorgei*. The bacteroids in mature *Dimorphandra* nodules were enclosed in plant cell wall material containing a homogalacturonan (pectic) epitope that was recognized by the monoclonal antibody JIM5. Analysis of sequences of their *rrs* (16S rRNA) genes and their ITS regions showed that the five *D. wilsonii* strains, although related to SEMIA6099, may constitute five undescribed species of genus *Bradyrhizobium*, whilst their *nodD* and *nifH* gene sequences showed that they formed clearly separated branches from other rhizobial strains. This is the first study to describe in full the N_2_-fixing symbiotic interaction between defined rhizobial strains and legumes in the sub-family Caesalpinioideae. This information will hopefully assist in the conservation of the threatened species *D. wilsonii*.

## Introduction

The Leguminosae (Fabaceae) is the third largest family of dicotyledonous plants with around 19,000 species that are generally divided into three subfamilies: Caesalpinioideae, Mimosoideae, and Papilionoideae [1]. Although most members of sub-families Mimosoideae and Papilionoideae are able to associate with soil bacteria to form N_2_-fixing root (and occasionally stem) nodules, very few genera of the Caesalpinioideae are known to nodulate [2], Those few that do belong to the tribes Cassieae and Caesalpinieae [2]; in tribe Cassieae only *Chamaecrista* is known to be able to form nodules, but in tribe Caesalpinieae *Campsiandra*, *Chidlowia, Dimorphandra*, *Erythrophleum*, *Melanoxylon, Moldenhawera* and *Tachigali* have nodulating species [2], [3], [4], [5].

Infection of legume roots by nodulating bacteria (collectively termed rhizobia) and nodule development has been studied in detail for a very few species of papilionoid and even fewer mimosoid legumes, but there are no published reports on the infection of any caesalpinioid species [6]. There is also a paucity of information about caesalpinioid nodule development, but it is known that of the two general types of legume nodules, those with limited growth (determinate) and those that retain meristematic activity (indeterminate), all nodules so far studied from caesalpinioid legumes are indeterminate [7], [8]. In addition, it is also known that although in most papilionoid and all mimosoid species, rhizobia are usually released into membrane bound vesicles called symbiosomes [9], those in a few papilionoid and all nodulated caesalpinioid trees remain confined within modified infection threads termed “persistent infection threads” or “fixation threads” [6], [7], [10], in which it is assumed (but not yet demonstrated) that they fix N_2_.

In this context, the present study focuses on infection and nodulation of the rare caesalpinioid species *Dimorphandra wilsonii* Rizz., and its associated microsymbionts. The genus *Dimorphandra* contains 26 species, all of which are trees native to South America, and although it was known that 11 of these species are nodulated, prior to the present study the nodulation status of *D. wilsonii* was unknown [2]. Although most *Dimorphandra* species (*e.g. D. exaltata*, *D. jorgei* and *D. mollis*) are widespread and not considered to be threatened [11], some are highly endemic and becoming increasingly rare *e.g. D. wilsonii* is confined to seasonally dry tropical forests in highland areas of Minas Gerais state in the Brazilian Cerrado [12], and is one of a group of species whose habitat has been destroyed by agricultural development and clearing of areas for cattle-pasture and production of charcoal [13]. This has been graphically demonstrated in recent years by Rizzini and Matos Filho [14], who found only 18 individuals in Minas Gerais (19° 16′ S, 44° 24′ W), and by Fernandes *et al*. [15] who located another 21 adult individuals in forage areas on two farms in the same area. Therefore, due to the destruction of habitat, very localized area of occurrence, and isolation of its population in remnant areas of the Cerrado, *D. wilsonii* was included in the Red List of Threatened Species [16] in the category of critically at risk.

There are no published studies on nodulation or N_2_ fixation in any species of *Dimorphandra*, but there is some information about their potential rhizobial symbionts. Rhizobia belong to the phylum *Proteobacteria*, mostly in the alpha class (e.g. *Rhizobium*, *Bradyrhizobium*), but some in the beta class (e.g. *Burkholderia*) [2], [17], [18], [19], and as with their infection and nodulation, relatively little is known about the rhizobia that nodulate caesalpinioid legumes. However, according to the currently available data *Bradyrhizobium* species may be their preferred endosymbionts [2], and in the specific case of *Dimorphandra*, two strains found in *D. jorgei* nodules, Br5004 (SEMIA6099) and Br5005 (SEMIA6400) [5], [20], were provisionally identified as *Bradyrhizobium elkanii* [21], [22], [23], as was a strain isolated from *D. parviflora* nodules [24]. However, it should be noted that (1) none of these strains were shown to nodulate their *Dimorphandra* hosts in these studies, and (2) that strains in other rhizobial species, such as *Rhizobium* and *Ensifer* (*Sinorhizobium*), have also been isolated from *Dimorphandra* nodules [24].

Therefore, the aims of this study are to: (1) determine the nodulation status of the rare legume *D. wilsonii* under natural and laboratory conditions, (2) to identify and characterize the rhizobia associated with *D. wilsonii*, and (3) to follow the infection and development of N_2_-fixing nodules on *D. wilsonii* roots by defined rhizobial strains, and to compare this with the infection of the widespread species, *D. jorgei*, by *Bradyrhizobium* strain SEMIA6099.

## Results

### Nodulation by Soil-grown and Inoculated *D. wilsonii*

**Figure 1:**
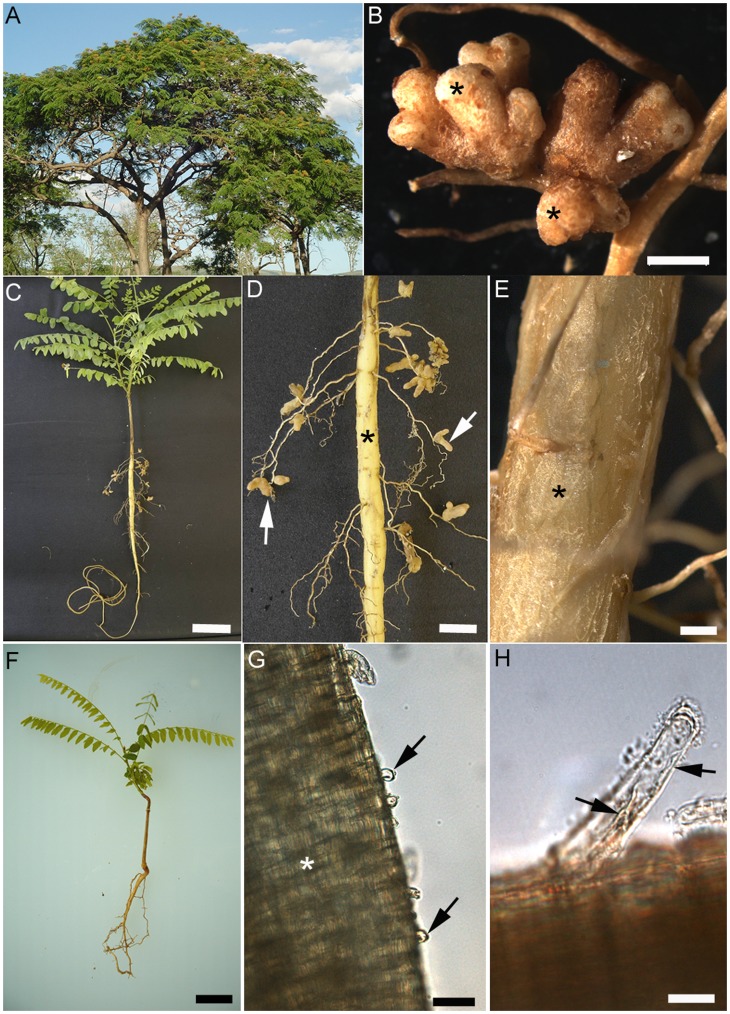
*Dimorphandra wilsonii*, a nodulated caesalpinioid legume native to Central Brazil. (A) *Dimorphandra wilsonii* tree growing in a Cerrado reserve at the Zoo-Botanical Garden, Belo Horizonte, MG, Brazil. (B) Nodules (n) from a *D. wilsonii* plant grown in a Leonard Jar using soil collected from the rhizosphere of adult trees. Twenty two rhizobial strains were isolated from nodules like these, tested for their ability to promote the growth of *D. wilsonii* under N-free conditions, and the five best performing strains (DW3.1, DW6.4, DW8.5, DW10.1, DW12.5) were selected for further examination. (C) Nodulated plant at 120 days after inoculation (dai) with rhizobial strain DW12.5. Note that the plant is green and healthy. (D) Detail of nodulated *D. wilsonii* root showing a swollen tap root (*), and large elongated nodules (arrows) on the fine lateral roots. (E) Detail of a swollen *D. wilsonii* tap root. Note the thick layer of spongy material (*). (F) Uninoculated *D. wilsonii* plant grown without any added nitrogen-containing fertilizer. This plant was harvested at the same time as that shown in (B). It had no nodules, and was showing signs of N-deficiency (i.e. yellowing leaves). (G) Light micrograph of short root hairs (arrows) on a *D. wilsonii* lateral root (*) at 42 dai with strain DW12.5. (H) Light micrograph of a longer root hair (arrow) at 42 dai with strain DW12.5. Note that it appears to contain an infection thread (IT) (arrows). Bars, 3 mm (B), 3 cm (C), 1 cm (D), 2 mm (E), 2 cm (F), 100 µm (G), 10 µm (H).

Soil was taken from the rhizosphere of nodulated *Dimorphandra wilsonii* growing in a reserve in the Cerrado (Figure 1A). “Trap” plants grown in the Cerrado soil were well nodulated, with large branched nodules (Figure 1B). Evidence for nodulation by *D. wilsonii* with specific rhizobial strains came from greenhouse experiments with plants that had been inoculated separately with five strains isolated from the nodulated trap plants. Inoculation with these strains resulted in nodulation of all the inoculated plants by the time that they were harvested at 120 days after inoculation (dai) (Figure 1C), with nodules forming on lateral roots (Figure 1D) that formed from a thick spongy tap root (Figure 1E). The average number of nodules per plant for each strain were 5±1.08 (DW12.5), 7±1.03 (DW3.1), 11±1.09 (DW6.4), 15±0.83 (DW10.1) and 18±1.17 (DW8.5). There were no significant differences between the strains in terms of number of nodules per plant: uninoculated plants had no nodules. The nodulated plants (Figure 1C) were also visibly greener and healthier than the non-nodulated control plants (Figure 1F), and at harvest were twice the height of the uninoculated plants (Figure 1C, F). Close examination of nodulated lateral roots at 60 dai revealed very short root hairs (Figure 1G), and some longer ones which appeared to contain infection threads (Figure 1H). No root hairs could be observed on the spongy tap roots (Figure 1E).

None of the *D. wilsonii* strains could nodulate soybean, with the exception of strain DW10.1 which produced an average of 5 nodules per plant. This compares with an average of 29 nodules per plant with *B. elkanii* 29W, a commercial strain used to inoculate soybean in Cerrado soils. The nodulation data were reflected in the plant dry weights, which, with the exception of plants inoculated with the commercial strain, 29W, were not significantly different to uninoculated controls (data not shown).

### Infection and Development of Nodules on *D. wilsonii* After Inoculation with *Bradyrhizobium* Strains

**Figure 2:**
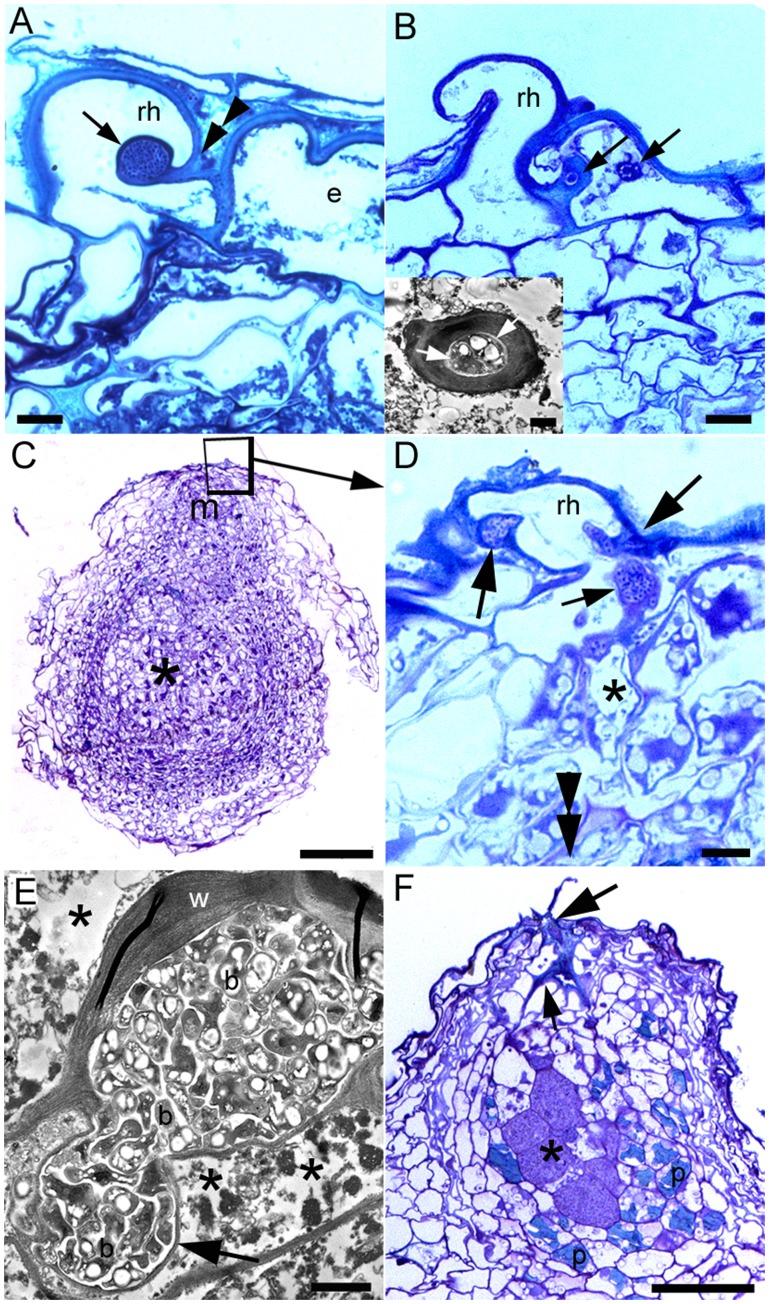
Light microscopy of toluidine blue-stained semi-thin sections (A–D, F) and transmission electron microscopy (TEM) of ultrathin sections (inset in B, E) illustrating the infection and development of nodules on the lateral roots of *D. wilsonii* at 42 dai with *Bradyrhizobium* strain DW10.1 (A, F) or DW12.5 (B–E). (A) Short root hair (rh) with an infection thread (IT) containing a large colony of bacteria (arrow). The IT appears to have originated at the inside of a curl in the root hair (double arrowhead). (B) Curled root hair (rh) and adjacent epidermal cell (e). The root hair itself does not appear to have been invaded, but the epidermal cell has been invaded by ITs (arrows, and inset) that appear to have emerged from the cell wall between the two cells. (C) Nodule primordium emerging from a lateral root. The point of infection is at the tip of the nodule (within the region delineated by a square), and is close to the nodule meristem (m). The spherical/oval-shaped main body of the nodule (*) consists largely of newly-infected cells. (D) Detail of the area in the square in panel D showing that the point of infection of the nodule in panel consists of a curled root hair (rh) being subjected to multiple invasions by bacteria (large arrows). These bacteria have then infected the adjacent cortical cells via enlarged ITs (small arrows), before progressing into the root through collapsed/collapsing cortical cells (*) towards the meristem of the emerging nodule primordium (indicated by a double arrowhead). (E) TEM of an enlarged IT in a collapsing root cortical cell located two or three cell layers deeper into the root and approaching the meristem of the emerging nodule (*i.e.* in the region marked * in panel D). Note that the enlarged IT contains numerous bacteria (b), and also that one side of it is bounded by a thick cell wall (w), whereas the other side has a much thinner cell wall, and appears to be releasing the bacteria into the degraded cytoplasm (*) of the host cell (arrow). (F) Emerging nodule primordium containing a small group of infected cells (*) surrounded by blue-green phenolic-containing cells (p). The remains of the original infection can be seen at the tip of the nodule primordium (large arrow), as can the progression of the infecting bacteria through the root cortex, the remains of which are a line of collapsed host cells (small arrow). Bars, 10 µm (A, B, D), 100 µm (C), 1 µm (B inset, E), 50 µm (F).

**Figure 3:**
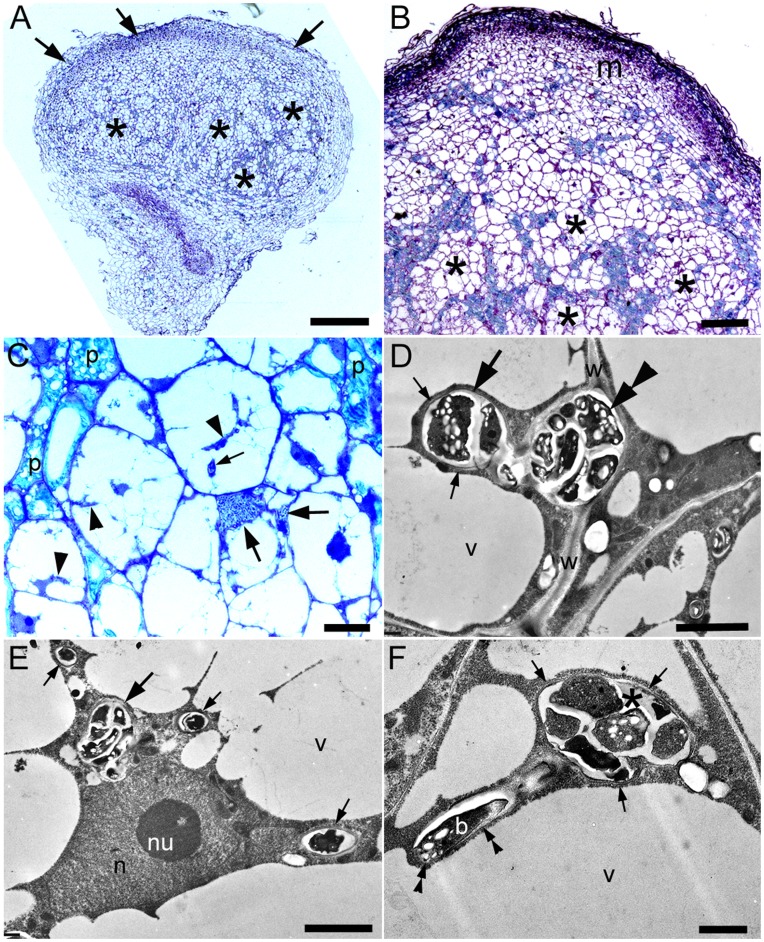
Light microscopy (A–C) and TEM (D, E, F) of developing (i.e. pre-N-fixing) nodules on a *D. wilsonii* root at 63 dai with *Bradyrhizobium* strain DW12.5. (A) The young nodules are spherical/oval in shape, but a broad apical meristem is still apparent (arrows), as would be expected in an indeterminate nodule. The remainder of the nodule is effectively an “invasion zone”, as it is being actively invaded by rhizobia (see subsequent panels), but there are no N-fixing cells containing bacteroids established at this stage. (B) The invasion zone behind the large meristem (m) is divided into “sectors” (*) by files of blue-green phenolic-containing cells. (C) High magnification view of a “sector” within the invasion zone that is delimited by phenolic-containing cells (p). There are groups of intercellular bacteria (large arrows), and these appear to be the origin of the bacteria in the IT that is infecting the adjacent cell (small arrow). Strands of cytoplasm containing bacteria are indicated by arrow heads. (D) Intercellular bacteria (double arrowhead) within the thick cell walls (w) between two cells; note that an invasive IT (large arrow) has branched from the intercellular bacteria and has started to invade the more vacuolated of the two adjacent cells. Also note the relatively thin cell wall surrounding this invasive IT (small arrows). (E) Cell in a “sector” containing intracellular ITs, the largest of which (large arrow) is associated with the host cell nucleus. The narrower ITs (small arrows), which may have originated from the large one, are contained within thin strands of host cytoplasm. (F) A large intracellular IT (*) that is in the process of releasing bacteria (b) into a host cell. Note that the IT has a cell wall (arrows), as does the bacterium that is in the process of being “released” (double arrowheads) into a thin strand of cytoplasm. n, nucleus; nu, nucleolus; v, vacuole. Bars, 500 µm (A), 100 µm (B), 10 µm (C), 2 µm (D, E), 1 µm (F).

**Figure 4:**
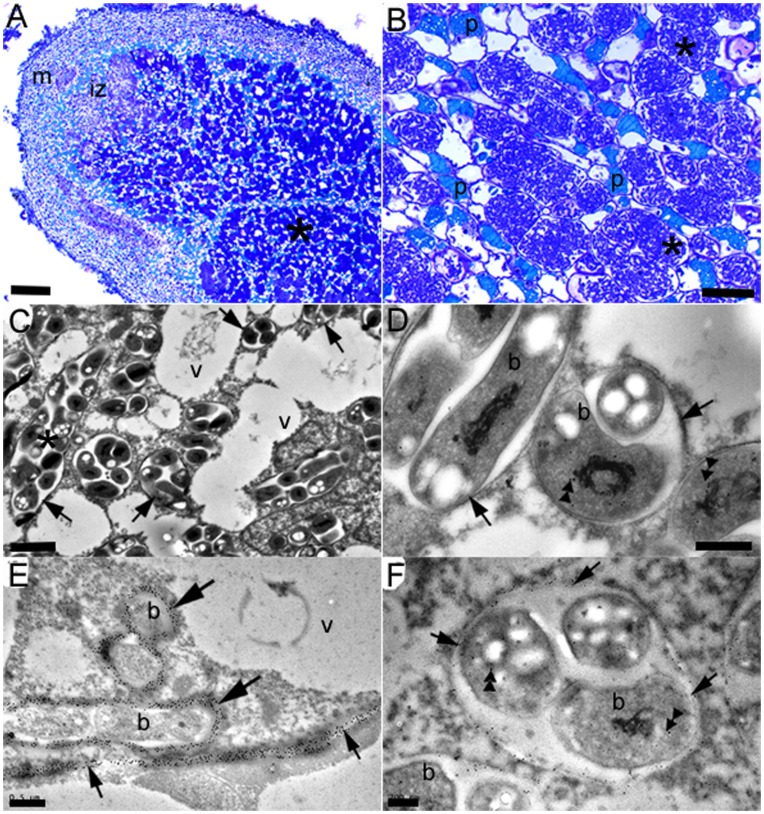
Light microscopy (A, B) and TEM (C–F) of mature, N-fixing *D. wilsonii* nodules at 63 dai with *Bradyrhizobium* strains DW12.5 (A–C, E) or DW10.1 (D, F). (A) Longitudinal section of a lobe from a large branched nodule shows the typical zonation of an indeterminate nodule, with an apical meristem (m), an invasion zone (iz) and a large infected N-fixing zone (*). Note the file of uninfected cells in the infected zone that contain phenolic compounds (arrow). (B) High magnification light micrograph of the infected zone shows the infected cells (*) to be filled with persistent infection threads (PITs), and to be interspersed with uninfected, phenolic-containing cells (p). (C) More detail of the infected cells can be seen under the TEM, where groups of bacteria, up to 15 per symbiosome (*), are enclosed in cell walls (arrows). v = vacuole. (D) Immunogold labeling of the nodules with an antibody against the *nifH* protein (Fe-protein) shows that the bacteroids (b) within the PITs/symbiosomes express this essential component of the nitrogenase enzyme complex (double arrowheads). Note the cell wall material surrounding the symbiosomes (arrows). (E) Infection threads in the invasion zone; note that the walls of these are strongly immunogold labeled with JIM5 (large arrows), a monoclonal antibody that recognizes a homogalacturonan epitope in pectin, which is a major component of plant cell walls. Note that the cell wall separating the infected cell from its neighbor is also strongly labeled (small arrows). b = bacteria, v = vacuole. (F) Persistent infection threads within the N-fixing zone: the walls of these are also immunogold labeled with JIM5 (arrows), thus confirming that the bacteroids (b) which are expressing nifH protein (double arrowheads) are also surrounded by cell wall material. Note, however, that the JIM5-labeled PIT walls are considerably thinner than those enclosing the invasive infection threads in (E). Serial sections to (D, E, F) that were treated with non-immune serum substituted for either the nifH protein or JIM5 antibodies showed no labeling (not shown). Bars, 200 µm (A), 20 µm (B), 2 µm (C), 500 nm (D, E), 200 nm (F).

**Figure 5:**
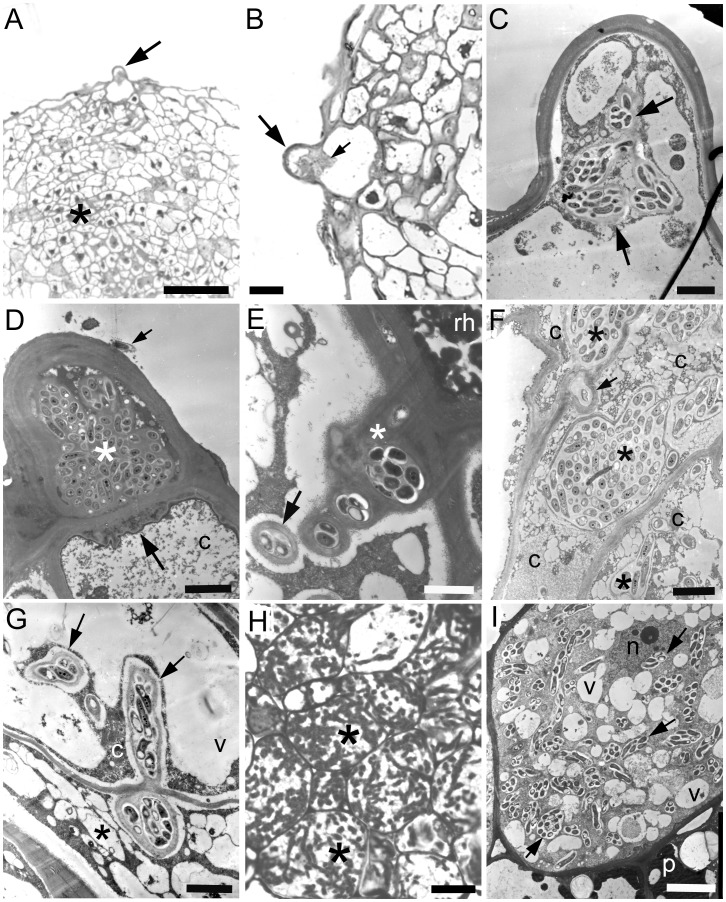
Light microscopy (A, B, H) and TEM (C–G, I) of the infection and development of N-fixing nodules on *D. jorgei*. These micrographs are of samples taken from nodulated roots at 60 dai with *Bradyrhizobium elkanii* strain SEMIA6099. (A) Root hair (arrow) at the tip of a nodule primordium consisting of newly-divided cells (*). (B) Higher magnification of the root hair (large arrow) showing that it appears to have been infected (small arrow). (C) Detail of the root hair confirming that it has been infected with bacteria; note that the bacteria are surrounded by a thick cell wall (arrows). (D) A root hair that is almost completely filled with bacteria that are embedded in an electron-dense matrix (*). There is a bacterium attached to the surface of the root hair (arrow), and note that the protrusion on the cell wall that is common to the adjacent cortical cell (c) appears to be the precursor of an invagination by the bacteria in the root hair into the cortical cell (large arrow). (E) Penetration of a cortical cell by an IT that has emerged from the cell wall (*) between the cortical cell and the adjacent root hair (rh); bacteria have been released from the IT and they are surrounded by a thick cell wall (arrow). (F) Cortical cells that have been infected by large groups (“pockets”) of bacteria that are surrounded by cell wall material (*). The large cell in the center of this micrograph has been infected via an IT (arrow) that has originated from a bacterial pocket in the adjacent cell at the top of the micrograph. Note that the host cytoplasm (c) is degraded in all the host cells, and that the cell in the center is shrunken in appearance. (G) Infection of a newly-divided nodule primordium cell by an IT (large arrow) that has entered from an adjacent degraded cortical cell (*). The newly-infected cell is highly vacuolated (v), but the invading IT has been surrounded by intact cytoplasm (c). (H) Mature, N-fixing cells packed with PITs (*). (I) TEM showing detail of an infected cell containing PITs with 5–10 bacteroids in each (arrows). The host cell is clearly viable, as can be seen by an intact nucleus (n). Note the numerous small vacuoles (v), and the adjacent cell which contains electron-dense phenolic material (p). Bars, 50 µm (A), 10 µm (B, H), 2 µm (C, D, F, G), 1 µm (E), 5 µm (I).

The infection and development of nodules on *D. wilsonii* is shown in Figure 1–4), and that of *D. jorgei* in Figure 5. No root hairs were observed on any of the inoculated or uninoculated plants until 42 dai, at which time small root hairs (Figure 1G, H), some containing infection threads (ITs) could be seen on lateral roots of *D. wilsonii* inoculated with either *Bradyrhizobium* strain DW10.1 or DW12.5 (Figure 1H, 2A–D). Small swellings close to the infected root hairs on these roots were shown by light microscopy and TEM to be developing nodule primordia (Figure 2C). Under laboratory conditions, the infection of *D. wilsonii* roots with either strain DW10.1 or DW12.5 thus appears to commence at some point just a few days prior to 42 dai, and coincides with the first appearance of root hairs. The infection process clearly involves the invasion of root hairs and/or adjacent epidermal cells, and the subsequent formation of ITs within them (Figure 2A–D). After this initial epidermal infection the bacteria progress further into the root cortex towards the emerging nodule meristem by invading the cortical cells in a somewhat “disorganized” manner via swollen ITs (Figure 2D, E); these thick-walled ITs move deeper into the root cortex, cell by cell, releasing bacteria into the cortical cells in thin-walled infection “pockets” (Figure 2E). The cortical cells thus invaded do not appear to tolerate the presence of such large numbers of bacteria (even though the bacteria are surrounded by cell wall material), and begin to senesce, as evidenced by degraded cytoplasm and the lack of any obvious organelles (Figure 2E). The infection continues into the adjacent cortical cell via ITs arising from the mass of wall-bound bacteria (not shown for *D. wilsonii*, but can be seen in *D. jorgei*; Figure 5F). The remnants of the infection process can be seen in some older nodules as a line of collapsed cortical cells connecting the original point of infection with the developing nodule (Figure 2F), which interestingly, at this stage is surrounded by uninfected cells that contain material that stains blue-green with toluidine blue, and which owing to these staining properties can thus be loosely interpreted as containing “phenolic” substances [25] or tannins [26].

Nodules at various stages of development were observed by 63 dai, ranging from nodule primordia similar to those seen at 42 dai, to mature nodules capable of expressing nitrogenase protein (Figure 3, 4). Smaller (*i.e.* younger) nodules were generally spherical or oval in morphology, but with a broad apical meristem (Figure 3A). A network of uninfected cells containing phenolic compounds could be observed throughout young nodules, and these cells appeared to delimit “sectors” of 20–30 newly-formed and highly vacuolated cells that were in the process of being infected by rhizobia (Figure 3A, B). In some cases, greatly enlarged intercellular ITs/infection pockets similar to those in Figure 2D, E were observed within these groups of cells (Figure 3C), and cells adjacent to these bacteria were being infected via intercellular ITs (Figure 3C, D). In other groups of cells, intercellular ITs were not obviously present, but the cells contained intracellular ITs adjacent to host cell nuclei (Figure 3E). Some of these ITs were observed releasing rhizobia into thin strands of host cytoplasm but the “released” rhizobia were always observed surrounded by cell wall material, which was quite thin at this early stage of development (Figure 3F).

Structurally, the mature nodules at 63 dai were effective in appearance and typical of nodules on other caesalpinioid legumes [7], [10] *i.e.* indeterminate with an apical meristem, an invasion zone, and an elongated central N_2_-fixing zone (Figure 4A) consisting of both infected cells and uninfected cells, with many of the latter containing phenolic compounds and/or tannins (Figure 4B, C). The infected cells were larger than the uninfected cells, and they were packed with bacteroids within persistent infection threads (“PITs”) of cell wall material (Figure 4B, C). The bacteroids within the PITs were immunogold labeled with an antibody against the *nifH* protein (Fe-protein), thus indicating that they were expressing this essential component of the nitrogenase enzyme complex (Figure 4D). Serial sections incubated in non-immune serum substituted for the nitrogenase antibody had no immunogold labeling (data not shown). Nodules were also examined using a monoclonal antibody (JIM5) that recognizes a homogalacturonan epitope in the pectic component of plant cell walls, and which has previously been shown to strongly label the wall of ITs in nodules on pea, as well as other papilionoid legumes [27]. In the present study, JIM5 also labeled invasive ITs very strongly (Figure 4E), but in contrast to pea nodules [27], it also labeled the symbiosomes, specifically the thin walls of the PITs surrounding the bacteroids (Figure 4F). It should be noted, however, that the labeling of the PIT walls was less intense than that observed on the thicker walls of the invasive ITs (Figure 4E).

Very similar data were obtained with *D. jorgei* plants inoculated with *Bradyrhizobium* strain SEMIA6099 (BR5004), and the whole developmental process could be observed by 60 dai. First, root hairs adjacent to developing nodule primordia were infected (Figure 5A, B) and massively colonized by the bradyrhizobia (Figure 5C, D). The bacteria within these then invaded cortical cells via ITs (Figure 5E), within which they established large, wall-bound “pockets”, the presence of which appeared to result in the senescence and collapse of the host cells (Figure 5F). However, as with *D. wilsonii* (Figure 3), the newly-divided cells in young *D. jorgei* nodules were able to receive the invasive ITs without them triggering host cell senescence (Figure 5G); the ITs subsequently released their rhizobia into the host cell cytoplasm, but they remained bound in plant cell wall material (Figure 5G). More mature nodules showed that the bacteria filled the host cells in the infected zone (Figure 5H), and that as with *D. wilsonii* they were enclosed in cell wall-bound PITs within which they were presumed to have fixed N_2_ (Figure 5I).

### Identification and Molecular Characterization of *D. wilsonii* Rhizobia via their *rrs* Gene (16S rRNA) and Internal Transcribed Spacer (ITS) Sequences

**Figure 6:**
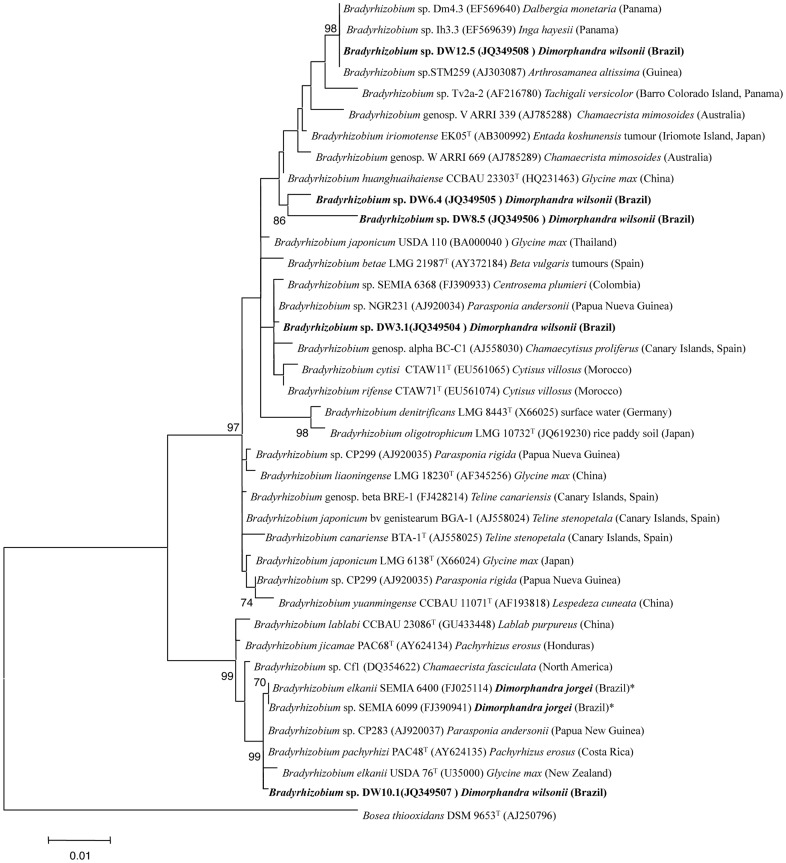
Maximum likelihood phylogenetic tree based on *rrs* (16S rRNA) gene sequences (1348 nt) showing the position of strains isolated from *Dimorphandra wilsonii* nodules within the genus *Bradyrhizobium*.

The phylogenetic analysis of the *rrs* gene of the five strains isolated from *Dimorphandra* nodules showed their affiliation to genus *Bradyrhizobium*, forming five independent branches (Figure 6). Four of the five *Dimorphandra* strains examined in this study (DW3.1, DW6.4, DW8.5, DW12.5) clustered into *Bradyrhizobium* group I, and the remaining strain, DW10.1, was located within *Bradyrhizobium* group II (both groups being originally defined by Menna *et al.* [23]).

*Bradyrhizobium* group I encompasses phylogenetically close species with similarities ranging from 98.4%, between the type strains of *B. iriomotense* and *B. denitrificans*, to 99.6%, between *B. japonicum* and *B. liaoningense*. To this group also belong some strains isolated from the caesalpinioid species *Chamaecrista mimosoides* [28] and *Tachigali versicolor* [29], as well as from the non-legume *Parasponia* [30]. None of them were the closest relatives to those of the *Dimorphandra* bradyrhizobial strains (Figure 6). Strain DW3.1 grouped within a wide cluster that also contained the type strains of the recently described species *B. rifense* CTAW71^T^ [31] and *B. cytisi* CTAW11^T^, the strain BC-C1 of genospecies alpha, as well as other strains isolated from different hosts from diverse geographical locations. The closest relatives to DW3.1 were the strains NGR231 isolated from *Parasponia* (99.9% identity) and SEMIA6368 (99.8% identity). Also the type strains of *B. cytisi* CTAW11^T^ and the recently described *B. rifense* CTAW71^T^ were phylogenetically close with identity values higher than 99.5%. The other three *D. wilsonii* strains within *Bradyrhizobium rrs* gene group I (DW6.4, DW8.5 and DW12.5) formed three independent branches within a wide cluster that included several strains isolated from various hosts from several geographical locations (Figure 6). The similarities amongst the *D. wilsonii* strains were lower than 98%, with 96.5% between DW12.5 and DW8.5, 97.6% between DW6.4 and DW8.5, and 97.5% between DW6.4 and DW12.5. These strains also showed identity values lower than 98.8 and 99.3% with respect to the two defined species included in this cluster, *B. iriomotense* EK05^T^ [32] and *B. huanghuaihaiense* CCBAU 23303^T^ [33], respectively. Considering that many different species comprising *Bradyrhizobium* group I showed more than 99.5% similarities in their *rrs* gene sequences, on the basis of this gene the *D. wilsonii* strains DW6.4, DW8.5 and DW12.5 could represent three novel species of *Bradyrhizobium*.

**Figure 7:**
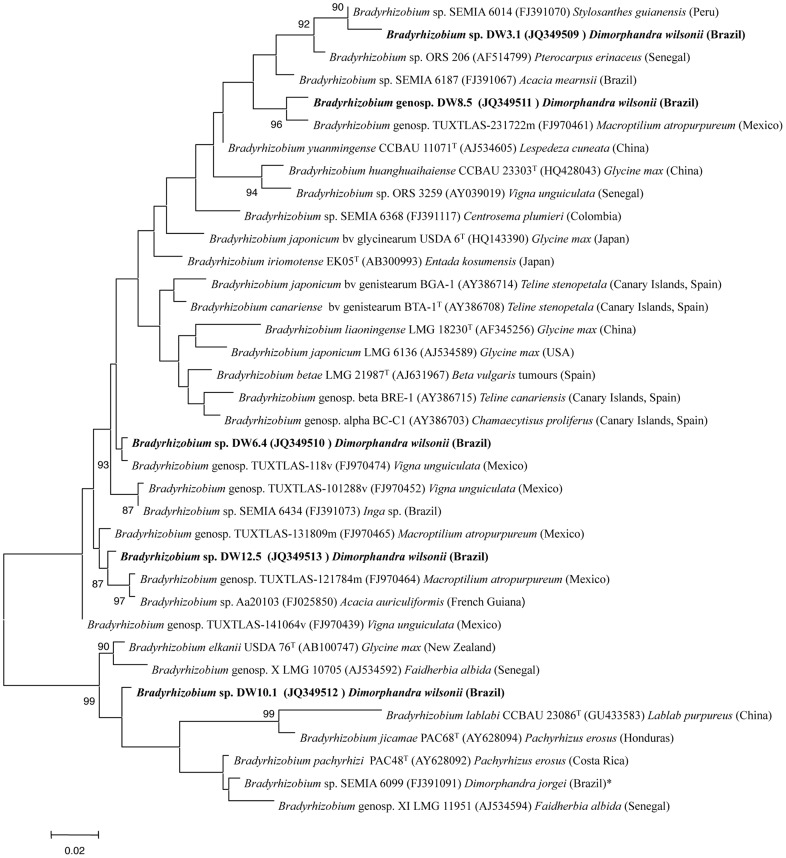
Maximum likelihood phylogenetic tree based on 16S-23S rDNA intergenic spacer (ITS) sequences (604 nt) showing the position of strains isolated from *Dimorphandra wilsonii* nodules within the genus *Bradyrhizobium*. Bootstrap values were calculated for 1000 replications. Bar, 1 nt substitution per 100 nt.

Only one strain examined in this study, DW10.1, clustered within the *Bradyrhizobium rrs* gene group II, a group in which two strains (SEMIA6400 and SEMIA6099) previously isolated from *Dimorphandra jorgei* are also located [22], [23]. To this group also belongs the strain Cf1 isolated from the caesalpinioid species *Chamaecrista fasciculata* [34] and the strain CP283 isolated from the non-legume *Parasponia andersonii* [30], with the latter being more closely related to strains from *Dimorphandra* than the *C. fasciculata* strain (Figure 6). The identities of the three bradyrhizobial strains isolated from *Dimorphandra* species (DW10.1, SEMIA6400, SEMIA6099) ranged between the type strains of *B. jicamae* and *B*. *elkanii* (99.5%) and the type strains of *B. elkanii* and *B. pachyrhizi* (100%). Indeed, strain DW10.1 showed 100% 16S rRNA sequence identity with respect to the type strains of *B. elkanii* and *B. pachyrhizi*, but identities of 100% (or close to 100%) are common among species of *Bradyrhizobium*, and they can only be differentiated on the basis of their ITS sequences (which are linked in most cases to DNA-DNA relatedness; [35]). According to the results of the ITS analysis, *Bradyrhizobium* species are distributed into two groups coincident with those found after *rrs* gene analysis (Figure 7). Also in agreement with the results of the *rrs* gene sequences, the strains from *Dimorphandra* are located in five branches, with strains DW3.1, DW6.4, DW8.5 and DW12.5 belonging to the ITS group 1, and DW10.1 to ITS group 2.

Strains DW3.1 and DW8.5 belong to the same ITS subgroup (Figure 7) that also contains strains isolated in different geographical locations from various hosts (see Figure 7). The internal identities of this subgroup, calculated after pairwise analysis and without gaps, are low, with 96.4% identity, between strains DW8.5 and TUXTLAS-231722 m isolated from siratro (*Macroptilium atropurpureum*) [36] and 96.3% between DW3.1 and SEMIA 6014, which was isolated from *Stylosanthes* (96.3% identity). They have 93.2 and 94.1% identity, respectively, to the type strain of *B. yuanmingense,* their closest relative in terms of described *Bradyrhizobium* species. Considering that strains from the same *Bradyrhizobium* species with more than 95% identity in the ITS fragment are in the same species [35], DW3.1 thus most probably represents an undescribed species of *Bradyrhizobium*, as does strain DW8.5.

Rhizobial strains DW6.4 and DW12.5 were the most closely related of the five *D. wilsonii* strains on the basis of their *rrs* gene sequences (Figure 6), but as their ITS sequences are only 93.2% identical they probably belong to different species. Indeed, they both cluster in different ITS subgroups, in which no described *Bradyrhizobium* species are present (Figure 7). However, both subgroups contain other strains that were isolated in the Americas, including strain 8v from genospecies TUXTLAS-11 isolated from *Vigna* in N. America [36], which probably is the same species as DW6.4, as their ITS sequences are 99.1% identical. Strains Aa20103 and 1784 m from genospecies TUXTLAS-12 [36] are closely related to DW12.5, with 98.1% and 98.0% identity values, respectively, and it is, therefore, likely that these three strains belong to the same species. These two putatively novel species (one including strains DW6.4 and 8v, and the other one containing strains DW12.5, Aa20103 and 1784 m) are also very different to those currently described in genus *Bradyrhizobium*, since the closest relative species are *B. iriomotense* and *B. yuanmingense* (branching in other ITS subgroups), with identity values of 93.9 and 93.8%, respectively, in relation to strain DW6.4, and 91.8 and 92.5%, respectively, in relation to strain DW12.5 (Figure 7).

The *D. wilsonii* bradyrhizobial strain DW10.1 (which is in *rrs* gene group II) belongs to an ITS subgroup supported by a bootstrap of 99% that includes the type strains of *B. elkanii* USDA 76^T^, which was isolated from soybean nodules, *B. lablabi* which was isolated from *Lablab purpureus* [37], and *B. pachyrhizi* PAC48^T^ and *B. jicamae* PAC68^T^, which were isolated from *Pachyrhizus* nodules [38]. Strains from the genospecies × and XI of [35] are close to the aforementioned strains, and together with them they form a wide group that also contains strain SEMIA 6099 which was isolated from *Dimorphandra jorgei* [5] and which has an identity value of 94.4% with respect to DW10.1. Interestingly, although SEMIA 6099 (together with strain LMG 11951 from genospecies XI), should be classified as *B. pachyrhizi*, as they have identity values higher than 98.4%, strain DW10.1 may actually belong to a separate species, as it only has 95.5% identity with respect to *B. elkanii* USDA 76^T^, which is at the limit for species differentiation in bradyrhizobia [35].

### Phylogeny of Symbiosis-related Genes (*nifH* and *nodD*) from *D. wilsonii* Rhizobia

The *nodD* and *nifH* genes were sequenced in the same strains used to study the infection process in *D. wilsonii*, *Bradyrhizobium* sp. DW10.1 and *Bradyrhizobium* sp. DW12.5, both clustering into different groups based on *rrs* and ITS phylogenetical analyses. Analysis of the *nodD* gene showed that both strains belong to two different lineages with only 75.3% identity between them (Figure S1). The closest related type strain to both strains DW10.1 and DW12.5 was *B. jicamae* PAC68^T^, isolated from *Pachyrhizus erosus* in Honduras (Central America), with 78.9 and 76.6% similarities, respectively.

As with their *nodD* sequences the *nifH* genes of strains DW10.1 and DW12.5 were divergent (although much less so than their *nodD* genes), with only 86.3% similarity between them (Figure S2). The closest related type strain to DW10.1 again was *B. jicamae* PAC68^T^ with 89.5% identity, but other unclassified strains had higher *nifH* similarities, such as two Chinese strains isolated from *Lablab* (CCBAU 61434) and *Arachis* (CCBAU 23160) both with 99.1% identity. The *nifH* gene of strain DW12.5 showed 87.8% identity with respect to *B. jicamae* PAC68^T^ and 86% identity with respect to the type strain of *B. canariense*, which was isolated in the Canary Islands from tagasaste (*Cytisus* [syn. *Chamaecytisus*] *proliferus*). In addition, CCBAU 65788 isolated in China from *Pueraria* had a *nifH* gene sequence closely related to that of DW12.5, with 91.8% identity (Figure S2). It is remarkable that the *nifH* gene of the bradyrhizobial strain Cf1 isolated from the caesalpinioid species, *Chamaecrista fasciculata* [34], was phylogenetically divergent from those of *Dimorphandra wilsonii* strains (Figure S2) and also from that of strain ANU 289 isolated from *Parasponia* [39].

## Discussion

### Infection and Development of Nodules on *D. wilsonii*

Five *Bradyrhizobium* strains (DW3.1, DW6.4, DW8.5, DW10.1 and DW12.5) that were isolated from *D. wilsonii* nodules were all shown to have the ability to nodulate their host under axenic N-free conditions (*i.e.* in sand/vermiculite-filled Leonard jars). The two *D. wilsonii Bradyrhizobium* strains that appeared to be the most effective at nodulation (DW10.1, DW12.5) were examined in more detail for their ability to infect and nodulate *D. wilsonii* seedlings under controlled conditions. The infection process of *D. wilsonii* by these strains was also compared with that of *D. jorgei* by another *Bradyrhizobium* strain, SEMIA6099, which was originally isolated from *D. jorgei* [5]. Both *D. wilsonii* strains were capable of inducing nodules on their host by 42 dai, and both of them infected *D. wilsonii* via root hairs that had formed on their lateral roots by this time. No root hairs were observed on either the thick spongy tap roots or on the lateral roots in the weeks prior to the 42 dai harvest. This is not unprecedented, as several woody legumes are known not to produce root hairs at all, and by definition these must nodulate through a non-root hair infection process [40]. However, in the case of *D. wilsonii*, although root hairs are actually produced, in our experimental set up their appearance was delayed until just a few days prior to 42 dai, and this delay would thus explain the initiation of nodules at such a relatively late stage after the seedlings were inoculated. The late formation of root hairs and the subsequent delayed development of nodules on *D. wilsonii* might be related to nutrient acquisition, as it is well known that the large seeds of legume trees contain substantial reserves of N (and other nutrients) [40], and thus there is less urgency for seedlings to initiate the nodulation process (or indeed to produce root hairs) immediately after germination.

In soybean and most other legumes so far examined with a root hair infection [9], [40], [41], rhizobia are conveyed from the hair to the nodule primordium through tubular, cell wall-bound ITs. The latter penetrate the newly-divided nodule cells, and the rhizobia are then released from the ITs into the host cytoplasm via unwalled infection droplets, upon which they are endocytosed by being immediately surrounded by a host-derived plasma membrane (the “symbiosome membrane”). Once enclosed within these “symbiosomes” the rhizobia then begin their transition into symbiotic N-fixing bacteroids. The symbiosome is effectively an organelle that creates an environment allowing for the optimization of N-fixation by the rhizobial bacteroids, and the symbiosome membrane mediates the efficient exchange of fixed N from the bacteroids for C-containing compounds from the host [42].

In the case of *Dimorphandra*, the steps in the *D. wilsonii* and *D. jorgei* nodulation processes were generally similar to those reported for crop legumes nodulated by *Bradyrhizobium*, such as soybean [41], rather than the processes involving crack entry and/or intercellular bradyrhizobia described for tagasaste [43] and *Lupinus albus* [44]. The main difference to the aforementioned process in soybean (and most other described legumes) is that the initial root hair infection was more “aggressive” in the *Dimorphandra* species. It also involved the relatively large scale and somewhat disorganized invasion of adjacent epidermal and cortical cells via large ITs, and the subsequent formation of large wall-bound pockets within these cells, which upon being invaded then senesced as the bacteria moved deeper into the root towards the developing nodule meristem via further enlarged ITs.

In common with other caesalpinioid legumes [7], [10], a few papilionoid legumes, but no mimosoid species [40], and with the non-legume *Parasponia* (family Cannabaceae in the order Rosales) [45], [46], [47], the rhizobia in *D. wilsonii* and *D. jorgei* nodules remain enclosed in host cell wall material after they are released into the newly-divided cells from the invasive ITs. These plant cell wall-bound structures are termed “persistent infection threads” (PITs) or “fixation threads” [40], [46], and this study has shown that in common with “normal” invasive ITs [27] the PITs in Caesalpinioid legumes are, indeed, surrounded by material that contains homogalacturonan epitopes (recognized by the monoclonal antibody JIM5) that are also present in the pectic middle lamella of plant cell walls [27]. The present study has also shown for the first time that the bacteroids within PITs are capable of expressing nitrogenase protein, of fixing atmospheric N_2_, and transferring the fixed N to the host. How this is achieved in PIT-containing legumes (and *Parasponia*) apparently without the complex biochemical and anatomical aspects of the highly evolved symbiosome membrane [9], [42], and with the additional barrier of a cell wall surrounding the bacteroids is a subject worthy of further investigation. Even though the JIM5-labeled walls of the PITs are considerably thinner than those of normal ITs, they are still likely to impede both the diffusion of both gases and solutes, and thus negatively affect the efficiency of the symbiosis, and so it is relevant to pose the question as to why bacteroids are enclosed in PITs in these symbioses? In the specific case of *Dimorphandra* spp. and bradyrhizobia, a clue to this might lie in the quasi-pathogenic infection process involving the senescence and collapse of invaded root cells (epidermal and cortical). This suggests that the relationship between the two partners in the symbiosis is not completely stable, and thus necessitates the invading rhizobia being kept enclosed in cell wall material at every stage of the nodulation process, even including when they are within the more “compatible” cells of the nodule itself. Interestingly, a similar “quasi-parasitic” relationship was recently decribed by Op den Camp et al. [47] for the interaction of some rhizobial strains with the non-legume *Parasponia andersonii*, which also forms a symbiosis with rhizobia that involves the bacteroids being enclosed in PITs [45], [46].

### *Dimorphandra* Symbionts are Genetically Distinct from Other Bradyrhizobia According to their *rrs* genes, ITS Regions and Symbiosis-related Genes

This study has shown clearly using *rrs* gene and ITS sequences that *D. wilsonii* strains, in common with the only other known characterised *Dimorphandra* isolates, SEMIA6400 and SEMIA6099 from *D. jorgei* [22], [23], belong to the genus *Bradyrhizobium*. Four of the *D. wilsonii* strains (DW3.1, DW6.4, DW8.5, DW12.5) belong to *Bradyrhizobium* group I, and the remaining one (DW10.1) to *Bradyrhizobium* group II (along with SEMIA6400 and SEMIA6099), as defined by Menna *et al.* [23] on the basis of *rrs* gene analysis. Interestingly, although both groups contain strains isolated from other caesalpinioid legumes none of the *D. wilsonii* strains was particularly closely related to them, or to strains from *Parasponia*, at least in terms of their *rrs* sequences.

By using both *rrs* and ITS sequences our study has been able to more precisely classify the *Dimorphandra* strains within the genus *Bradyrhizobium*, and has shown that each of the five strains isolated from *D. wilsonii* nodules may constitute different undescribed species. This is in spite of the fact that *D. wilsonii* strain DW10.1 has a *rrs* gene phylogenetically close to SEMIA6099 from *D. jorgei*, which has been identified as a *B. pachyrhizi* strain, and is because DW10.1 and SEMIA6099 have very divergent ITS sequences. Our study also contains the first published description of the symbiosis-essential regulatory *nodD* gene from symbionts of a caesalpinioid legume. The analysis of both *nodD* and *nifH* genes showed that the *D. wilsonii Bradyrhizobium* strains formed branches clearly separated from other rhizobial genera suggesting that vertical transmission plays a predominant role in the persistence of symbiotic genes in *Bradyrhizobium*, as was already indicated by studies on bradyrhizobia from other legumes [18], [48], [49], [50]. Interestingly, none of the *D. wilsonii* strains could nodulate soybean (except for strain DW10.1, albeit ineffectively), which suggests that they possibly have a high degree of specificity towards their host. This will be investigated further via analyses of their *nodA* genes and of their host range on various legumes known to nodulate with bradyrhizobia, including other caesalpinioid legumes.

Taken together, the phylogenetic analysis of housekeeping and symbiosis-related genes suggests a very high diversity of bradyrhizobia nodulating *Dimorphandra* in tropical seasonally dry ecosystems, such as the Brazilian Cerrado. Interestingly, the symbionts of *Mimosa* spp. that are endemic to the Cerrado, although they are *Burkholderia* and not bradyrhizobia as far as has been found to date, are also very diverse and particular to this environment [51]. Indeed, the poor soils, highly seasonal precipitation and fire-dominated ecology of the Cerrado are known to be major reasons for making it a hotspot for legume biodiversity [52] and, presumably also of their associated rhizobial symbionts [51]. Further research into other endemic legumes (in all three sub-families) from this biome should provide a lot of information about co-evolution between legumes and rhizobia in extreme environments that have promoted endemicity [52].

### Bradyrhizobia may be the Preferred Symbionts of Caesalpinioid Legumes

As well as providing detailed information about *Dimorphandra* symbionts, by comparing their sequences (as far as is possible) with those from rhizobia isolated from other caesalpinioids the present study has also added to our very sparse knowledge about the rhizobial symbionts of caesalpinioid legumes in general. All caesalpinioid nodulating genera, with the exception of *Chidlowia* and *Erythrophleum*, which are endemic to West Africa [4], and the large pantropical genus *Chamaecrista*, are endemic and/or native to South and Central America, with Brazil being a major centre of diversity [2], [5]. Amongst the nodulating genera in the Caesalpinieae, the present study has confirmed that *Dimorphandra* spp. are nodulated by *Bradyrhizobium* [21], [22], [23], [24], and bradyrhizobia have also been isolated from nodules on *Melanoxylon* [53], *Tachigali* [24], [29]. *Campsiandra* is possibly nodulated by *Burkholderia* (Mitchell Andrews, personal communication), but nothing is yet known about symbionts of *Moldenhawera*. In the case of the African Caesalpiniod genera, *Erythrophleum* is nodulated by *Bradyrhizobium* [4], but the symbionts of *Chidlowia* so far remain unidentified. Finally, with regard to *Chamaecrista* (Cassieae), although very little is known about what nodulates the 280 plus species in this large genus, among the various rhizobial types that have been isolated from *Chamaecrista* nodules, bradyrhizobia have been the most commonly isolated genus, as shown by studies in Australia [28], China [54], Senegal [48] and the USA [34], although not, so far in Brazil, which, interestingly, is the centre of radiation of the genus [24].

On the basis of the somewhat sparse evidence so far obtained, it would thus appear that caesalpinioid legumes may have a preference for *Bradyrhizobium*. Interestingly, bradyrhizobia are also the preferred symbionts of *Parasponia* [30], [48], which is the only non-legume known to form nodules with rhizobia, and which, like so many caesalpinioid legumes, such as *Dimorphandra* (this study), also encloses them in PITs [45], [46], [47]. It also appears that bradyrhizobia, which are very ancient symbionts of legumes [55], [56], are highly adaptable in terms of their hosts, being capable of forming symbioses with non-legumes (*Parasponia*), basal (and therefore, “primitive”) legumes, such as the Caesalpinioideae (this study), and with “advanced” papilionoid legumes, such as soybean, lupins, and *Aeschynomene* spp. Bradyrhizobia are also adaptable in their methods of infecting their hosts. Although they often utilize the “classical” root hair pathway, such as in the present study and in other woody legumes, such as the mimosoid species *Acacia mangium* [26], many bradyrhizobial strains are capable of infecting their hosts via more “exotic” routes, particularly through variations on “crack entry”, as has been shown for tagasaste [43], *Lupinus* [44] and *Aeschynomene* spp. [57].

### Concluding Remarks

This study is the first report to describe in full a symbiotic association between a caesalpinioid legume and defined strains of rhizobia, but as with papilionoid legumes, we cannot exclude the possibility that not all caesalpinioid legumes have an infection pathway similar to *Dimorphandra*, especially considering their separation both in taxonomic terms and in time of nodule evolution [58]. Nevertheless, it does suggest that basal legumes can be infected via root hairs, which is considered a relatively “advanced” form of infection [2], [6], and further studies with other nodulated caesalpinioid legumes are needed to determine how typical such a process is in this paraphyletic sub-family.

The five nodulating strains isolated from *D. wilsonii* nodules that were studied in detail may represent up to five new *Bradyrhizobium* species, and sequences of the symbiosis-related genes, *nifH* and *nodD* suggest that *D. wilsonii* symbionts may be unique amongst the bradyrhizobia. Indeed, this uniqueness could be related to the particular environment within which *D. wilsonii* has evolved i.e. the acidic and low nutrient soils of the seasonally dry savannah of Central Brazil (the “Cerrado”). Understanding more fully how its N-fixing symbiosis can form and operate in such a hostile environment as the Cerrado will assist in current and future attempts to conserve (and even reintroduce) this rare and threatened species.

## Materials and Methods

### Isolation and Testing of Rhizobia for Nodulation of *D. wilsonii*

A pot experiment was set up to “trap” the symbiotic bacteria nodulating *D. wilsonii*. Seeds of *D. wilsonii* that had been scarified with sulphuric acid for 60 min were incubated in a germination chamber (25°C) with a photoperiod of 12 h until they germinated. Pre-germinated seeds were then transplanted into pots filled with 2 kg of soil taken from the rhizosphere of 10 year old, nodulated *D. wilsonii* trees growing in a Cerrado site (Zoo-Botanical Garden, Belo Horizonte, MG, Brazil) (Figure 1A). A permit (#5244138) for collection of soil and plant material from this site for research was obtained from the Instituto Brasileiro do Meio Ambiente e dos Recursos Naturais Renováveis (IBAMA), and was provided by the Sistema de Autorização e Informação em Biodiverside (SISBIO). The soil was characterized as a red-yellow Latosol, and it had low levels of nutrients, especially of phosphorus [59]. The experiment was performed in a greenhouse without supplemental light or temperature. Any nodules formed were harvested at 220 d after planting for isolation of rhizobia according to Somasegaran and Hoben [60], resulting in 22 isolates that might potentially be rhizobia based upon their growth rates and colony morphologies. These 22 isolates were then tested for their ability to nodulate *D. wilsonii* seedlings planted in sterilized soil in Leonard jars [61] according to [59] using 1 mL of a liquid culture of each isolate per jar (10^7^ CFU mL^−1^). The plants were harvested at 120 dai, and their biomass was determined as shoot dry weight.

The five best performing rhizobial isolates in terms of biomass production (isolates 3.1, 6.4, 8.5, 10.1, 12.5) were then selected for further analysis of their nodulation and N_2_-fixing ability, but this time under N-free conditions in pots filled with sterile sand and vermiculite. The experimental design was completely randomized with six treatments, consisting of an uninoculated control plus five different bacterial strains, with 3 replicates per treatment. Plants were examined visually every 30 d for the presence of root hairs, and were harvested at 120 dai. In order to test the ability of these strains to nodulate a crop legume commonly grown in the same environment in the state of Minas Gerais a similar Leonard Jar experiment was set using a commercial soybean (*Glycine max* L.) variety (BRSMG 68 Vencedora). Plants inoculated by *B. elkanii* strain 29W were used as positive controls, and uninoculated plants served as negative controls. All plants were harvested at 60 d after inoculation.

### Light Microscopy and Transmission Electron Microscopy (TEM)

A microscopical examination of the infection of *D. wilsonii* roots by rhizobial strains 10.1 and 12.5 was undertaken. *Dimorphandra wilsonii* seeds were surface-sterilized and germinated for 7 d in moisture chambers at room temperature and darkness. For long-term greenhouse experiments, seedlings were transferred to 2.5 L pots containing a sterile vermiculite and sand mixture (1∶1 v/v). After 28 d growth, each plant was inoculated with 50 ml of a liquid suspension of strain 10.1 or strain 12.5 (OD_600 nm_ 0.5; 4×10^8^) prepared in N-free Fåhraeus medium [62]. Pots were watered as needed and fertilized once a week with Fåhraeus N-free complete nutrient solution. Plants were harvested at 42 and 63 dai, and intact roots and root-hair development, as well as any developing nodules, were examined using light microscopy and TEM according to previous studies [63], [64]. More detailed analyses to detect and localize in nodules the Fe-protein (*nifH* protein) of the nitrogenase enzyme complex by immunogold labeling coupled with TEM were performed using the methods described by dos Reis Junior *et al.* [65]. Furthermore, as it has long been considered that the PITs in caesalpinioid nodules are composed of plant cell wall material [7], [10], TEM and immunogold labeling was also employed to localize a homogalacturonan epitope of the important cell wall component pectin by using the monoclonal antibody JIM5 (a gift from Dr N.J. Brewin, John Innes Centre, UK) which was originally used to demonstrate that the walls of infection threads in pea (*Pisum sativum*) nodules contained pectin [27]. Given that the nifH protein antibody was raised in rabbits and the JIM5 antibody was raised in rats it was also possible to perform a “double-labeling” immunogold experiment on mature nodules to determine if bacteroids expressing nifH protein were also surrounded by pectic material. Serial sections that were treated with non-immune serum substituted for either or both the nifH and JIM5 antibodies acted as negative controls [65].

The infection and development of nodules on *D. jorgei* was examined using the same basic methods as *D. wilsonii*, but with a few modifications to the sampling procedure. Briefly, nodules at various stages of development, from barely macroscopically visible “bumps” on roots to fully mature nodules, were sampled from plants at 60 dai with *Bradyrhizobium* sp. strain SEMIA6099, and then fixed and embedded for light microscopy and TEM as per *D. wilsonii*.

### Genotypic Characterization of Rhizobia Nodulating *D. wilsonii*

The five isolates tested for nodulation of *D. wilsonii* and soybean were examined genetically by obtaining sequences of their *rrs* (16S rRNA), *nodD* and *nifH* genes as well as their 16S-23S internal transcribed spacer (ITS). The *rrs* gene of all five strains was amplified and sequenced according to Rivas *et al.* [66], and their 16S-23S rRNA ITS was amplified and sequenced as described by Willems *et al.* [35]. The *nodD* and *nifH* genes of strains were amplified with the primers described by Rivas *et al.* [67]; PCR conditions for both were preheating at 95°C for 5 min, 35 cycles of denaturing at 95°C for 1 min, annealing at 53°C for 1 min and extension at 72°C for 1 min, and a final extension at 72°C for 7 min. The sequences were obtained in an ABI3100 sequencer (Applied Biosystems Inc.) using a BigDye terminator v3.1 cycle sequencing kit as supplied by the manufacturer. The sequences obtained were compared with those from GenBank using the BLASTN program [68]. Sequences were aligned using the Clustal W software [69], and the distances were calculated according to Kimurás two-parameter method [70]. Phylogenetic trees were inferred using the maximum likelihood method [71], and the bootstrap analysis was based on 1000 resamplings. The MEGA 5 package [72] was used for all analyses. All identity values were calculated by pairwise analysis and gaps were not considered.

## Supporting Information

Figure S1 Maximum likelihood phylogenetic tree based on *nodD* gene sequences (519 nt) showing the position of strains isolated from *Dimorphandra wilsonii* nodules compared with other rhizobial strains isolated from nodules of different legumes. Bootstrap values were calculated for 1000 replications. Bar, 5 nt substitution per 100 nt.
(PDF)

Figure S2 Maximum likelihood phylogenetic tree based on the *nifH* gene (381 nt) showing the position of strains isolated from *Dimorphandra wilsonii* nodules compared with other rhizobial strains isolated from nodules of different legumes. Bootstrap values were calculated for 1000 replications. Bar, 2 nt substitution per 100 nt.
(PDF)
