# Correction: Nodulation in *Dimorphandra wilsonii* Rizz. (Caesalpinioideae), a Threatened Species Native to the Brazilian Cerrado

**DOI:** 10.1371/annotation/eba72e14-aebd-494f-8121-7eb9fd8da168

**Published:** 2013-06-04

**Authors:** Márcia Bacelar Fonseca, Alvaro Peix, Sergio Miana de Faria, Pedro F. Mateos, Lina P. Rivera, Jean L. Simões-Araujo, Marcel Giovanni Costa França, Rosy Mary dos Santos Isaias, Cristina Cruz, Encarna Velázquez, Maria Rita Scotti, Janet I. Sprent, Euan K. James

The lower panels in Figure 4 are out of focus. Please see the corrected figure at the following link: 

**Figure:**
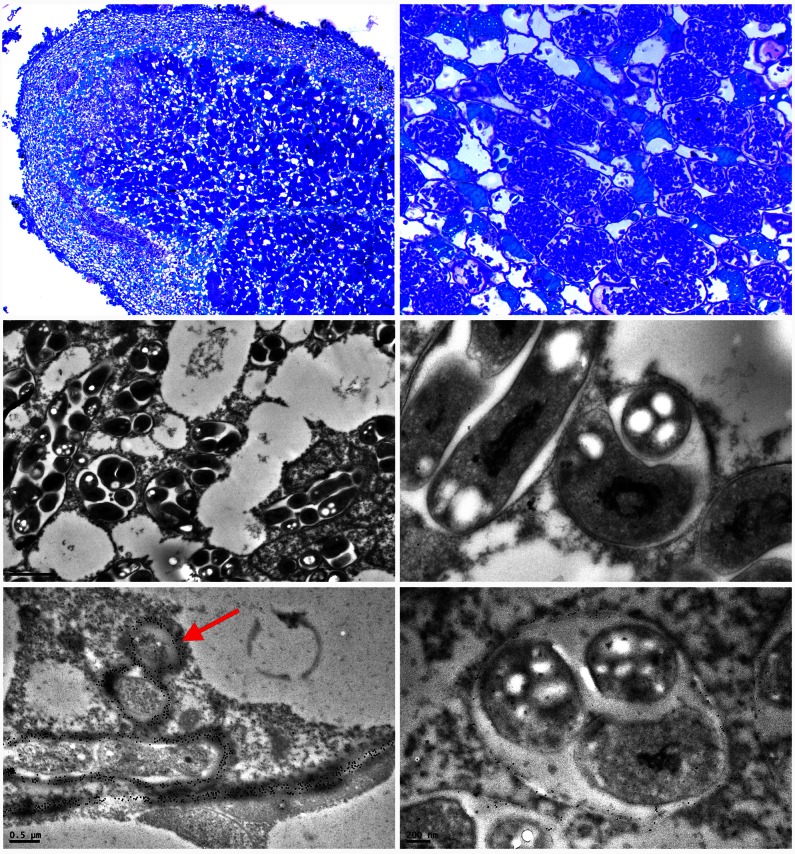


.

