# Supplementary figures and images for: Nodulation in Dimorphandra wilsonii Rizz. (Caesalpinioideae), a Threatened Species Native to the Brazilian Cerrado
Source: PLoS One. 2012 Nov 19;7(11):e49520. doi: 10.1371/journal.pone.0049520 (PMC3501520; doi:10.1371/journal.pone.0049520)

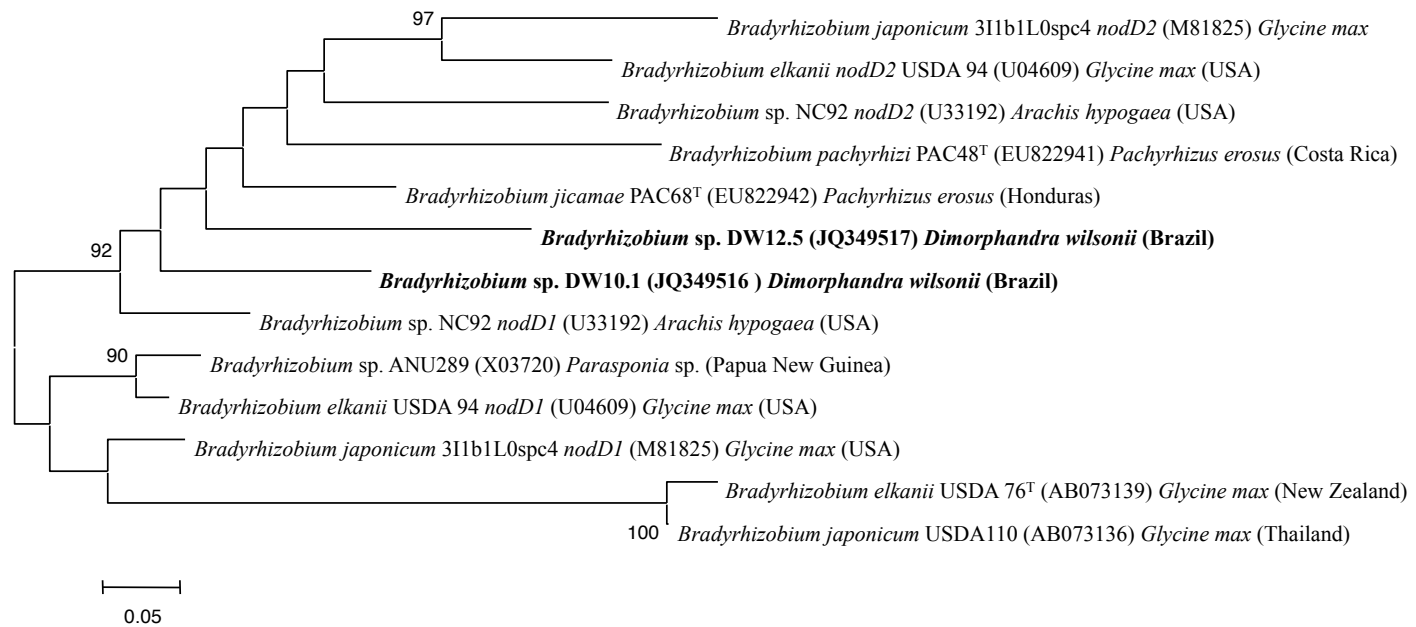

Supplement: Figure S1 — Maximum likelihood phylogenetic tree based on nodD gene sequences (519 nt) showing the position of strains isolated from Dimorphandra wilsonii nodules compared with other rhizobial strains isolated from nodules of different legumes. Bootstrap values were calculated for 1000 replications. Bar, 5 nt substitution per 100 nt. (PDF) [file pone.0049520.s001.pdf]

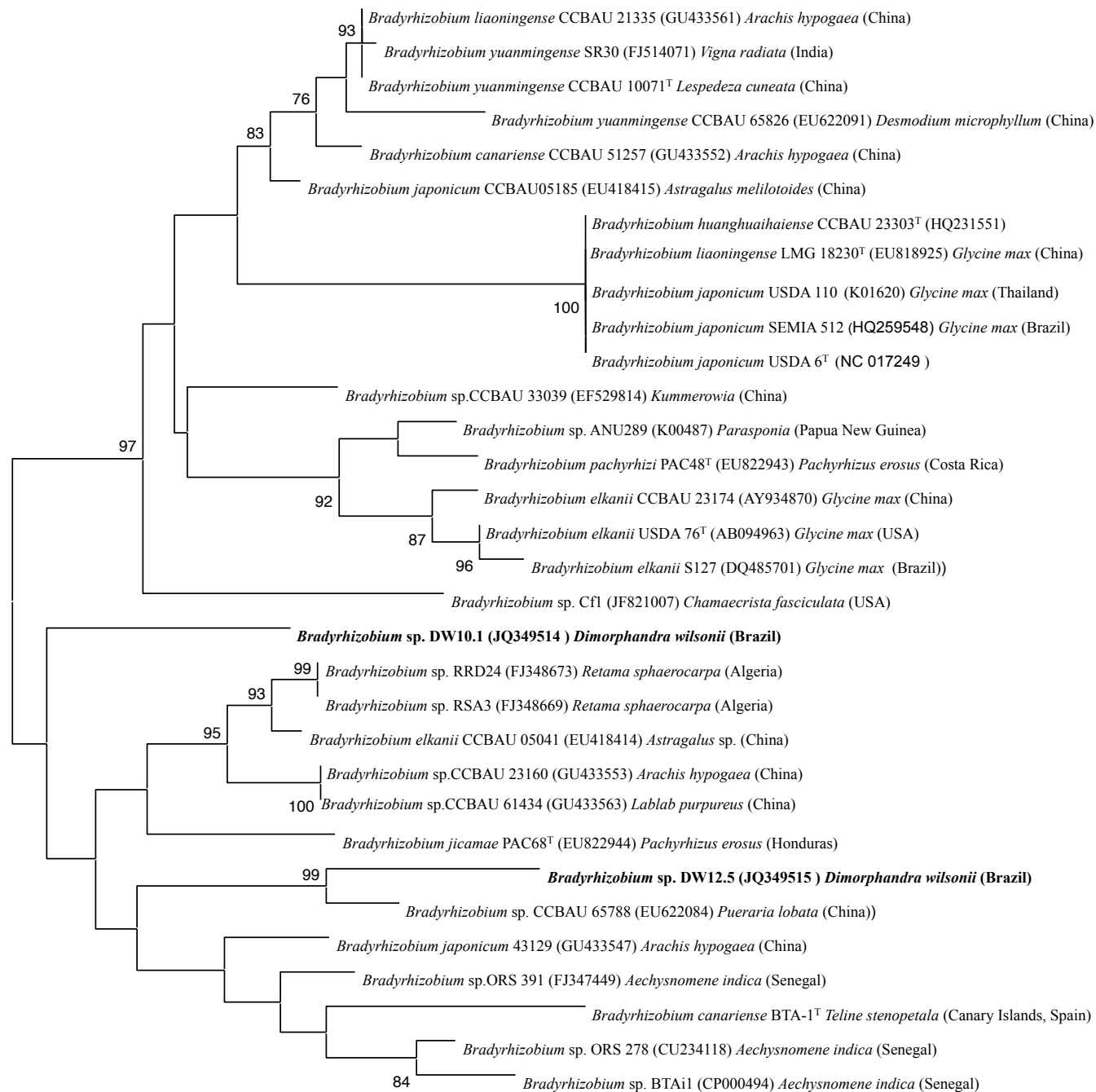

0.02

Supplement: Figure S2 — Maximum likelihood phylogenetic tree based on the nifH gene (381 nt) showing the position of strains isolated from Dimorphandra wilsonii nodules compared with other rhizobial strains isolated from nodules of different legumes. Bootstrap values were calculated for 1000 replications. Bar, 2 nt substitution per 100 nt. (PDF) [file pone.0049520.s002.pdf]
